# *N*-Glycomic Analysis of the Cell Shows Specific Effects of Glycosyl Transferase Inhibitors

**DOI:** 10.3390/cells10092318

**Published:** 2021-09-04

**Authors:** Qingwen Zhou, Yixuan Xie, Matthew Lam, Carlito B. Lebrilla

**Affiliations:** 1 Department of Chemistry, University of California, Davis, CA 95616, USA; qwzzhou@ucdavis.edu (Q.Z.); axexie@ucdavis.edu (Y.X.); mallam@ucdavis.edu (M.L.); 2 Department of Biochemistry, University of California, Davis, CA 95616, USA

**Keywords:** mass spectrometry, *N*-glycan, glycosyltransferase

## Abstract

Glycomic profiling methods were used to determine the effect of metabolic inhibitors on glycan production. These inhibitors are commonly used to alter the cell surface glycosylation. However, structural analysis of the released glycans has been limited. In this research, the cell membranes were enriched and the glycans were released to obtain the *N*-glycans of the glycocalyx. Glycomic analysis using liquid chromatography–mass spectrometry (LC–MS) with a PGC chip column was used to profile the structures in the cell membrane. Glycans of untreated cells were compared to glycans of cells treated with inhibitors, including kifunensine, which inhibits the formation of complex- and hybrid-type structures, 2,4,7,8,9-Penta-O-acetyl-*N*-acetyl-3-fluoro-b-d-neuraminic acid methyl ester for sialylated glycans, 2-deoxy-2-fluorofucose, and 6-alkynyl fucose for fucosylated glycans. Kifunensine was the most effective, converting nearly 95% of glycans to high mannose types. The compound 6-alkynyl fucose inhibited some fucosylation but also incorporated into the glycan structure. Proteomic analysis of the enriched membrane for the four inhibitors showed only small changes in the proteome accompanied by large changes in the *N*-glycome for Caco-2. Future works may use these inhibitors to study the cellular behavior associated with the alteration of glycosylation in various biological systems, e.g., viral and bacterial infection, drug binding, and cell–cell interactions.

## 1. Introduction

The glycocalyx is the carbohydrate component of the cell membrane composed of glycans on glycoconjugates such as protein and lipids. *N*-Glycans are bound to asparagine on proteins and make up potentially the largest component of the glycocalyx [1]. They are classified into three principal types: high mannose, complex, and hybrid types. High mannose structures are produced earlier in the glycosylation process, while complex- and hybrid-type structures are produced later [2,3]. Complex- and hybrid-type structures are further decorated by other monosaccharides, specifically, galactoses, fucoses, and sialic acids (*N*-acetyl-5-neuraminic acid or Neu5Ac). These additions play important roles in cell–cell interactions, including immune response, infection (viral and bacterial), and cell–cell adhesion [4,5,6]. The variations in compositions, linkages (with anomeric character), and regiochemistry can have profound effects on protein function. For example, the absence of core fucose is generally lethal in humans [7]. Similarly, sialic acids are important structures for binding viruses and bacteria leading to infection [8,9]. 

Another important function of glycosylation is regulating protein turnover rates. It has been shown that IgGs decorated with sialic acid have longer half-lives than those without it, suggesting that glycosylation can affect protein turnover rates [10,11]. We previously demonstrated the effect of glycosylation on protein turnover rates. In the study, we measured the turnover rate by feeding cells with isotope-labeled monosaccharide and measured the rate of incorporation of the exogenous sugars on the cell surface. It was shown that proteins glycosylated with high mannose had faster turnover rates than proteins with complex-type structures [12]. 

Past studies have shown that the use of mutations to induce defects in glycosylation was difficult or caused unpredictable outcomes [13,14]. The knockout of a specific glycosyltransferase can lead to another transferase taking over its role. Additionally, the removal of transferase genes, for example, FUT8, is lethal, making it more difficult to study in animals [15]. The development of metabolic glycosylation inhibitors helps overcome these limitations. Using metabolic glycosylation inhibitors is advantageous because small molecules are readily taken up into the cells. Additionally, these metabolic inhibitors make it possible to study animals since the amount needed to inhibit glycosylation is not lethal to the animals. These inhibitors enable the exploration of fundamental questions regarding the decoration of specific monosaccharides on glycans. For example, fundamental questions regarding the presence of fucose or sialic acids have been examined using inhibitors. It has been shown that the downregulation of fucose on glycans using 2-fluoro-l-fucose (2FF), a fucosyltransferase inhibitor, suppresses the proliferation and migration of the HepG2 cell. The study showed that 2FF targeted fructosyltransferase 8, which is responsible for core fucosylation [16]. Additionally, 6-alkynyl fucose (6AF) is another known fucose inhibitor. It inhibits the protein FX, which is responsible for converting mannose to fucose [17]. It has been known that inhibition with 6AF has halted cell migration and invasion in hepatoma cells [18]. The reduction of sialic acid using 2,4,7,8,9-Penta-*O*-acetyl-*N*-acetyl-3-fluoro-b-d-neuraminic acid methyl ester (3FS) causes the impairment of adhesion, migration, and in vivo tumor growth on the B16F10 cell. The sialic acid inhibitor is suspected to bind to sialytransferases after 3FS is converted to CMP-P-3FS [19]. Likewise, the increase in high mannose glycans using a mannosidase I inhibitor, Kifuensine (Kif), has been proven to increase the metastatic characteristics of CCA cells [20]. Though these inhibitors have been used in many studies, the alteration of the glycocalyx caused by the glycosylation inhibitor has not been well characterized using newer glycomics methods. Additionally, the effects on the expression of proteins in the glycocalyx by the presence of these inhibitors are little known.

The recent and general interest in the function of the glycocalyx has led to new tools to characterize them [21,22]. Within the last decade, nano-Liquid Chromatography–Mass Spectrometry (nanoLC–MS)-based methods have been developed to characterize the glycocalyx. Using nanoflow liquid chromatography is advantageous because its high sensitivity allows for the detection of low abundant glycans. The coupling with mass spectrometry allows structural characterization using fragmentation patterns. One method we developed to study the glycome on the cell surfaces uses a nanoflow liquid chromatography–quadrupole time-of-flight MS (nanoLC–QTOF MS). These methods have already been used to study glycans on human milk oligosaccharides as well as free glycans in bodily fluids [23]. 

In this research, we employed inhibitors to affect the cell surface glycosylation. We used N-glycans to monitor these changes and quantitate the change expressed by the inhibitors. The change caused by inhibition was monitored on three different cell lines (Caco-2, A549, and PNT2), and the changes in *N*-glycan profile were performed on a nanoLC-chip–QTOF MS. Additionally, using a nanoLC–Orbitrap MS, we performed proteomic analysis on Caco-2 to monitor the changes in protein expression that may be caused by altering the cell surface glycosylation.

## 2. Materials and Methods

### 2.1. Materials

Caco-2 cell lines, A549 cell lines, and Eagle’s Minimum Essential Medium (EMEM) were obtained from the American Type Culture Collection (ATCC), Manassas, VA, USA. Dithiothreitol (DTT), iodoacetamide (IAA), and a human immortalized prostate PNT2 cell line were purchased from Sigma-Aldrich, St. Lousi, MO, USA. Phosphate-Buffered Saline (PBS), Roswell Park Memorial Institute (RPMI) 1640 medium, fetal bovine serum (FBS), and penicillin were purchased from ThermoFisher Scientific, Waltham, MA, USA. Sequencing Grade Modified Trypsin was purchased from Promega. Glycosylation inhibitors Kifunensine, 2-deoxy-2-fluoro-l-fucose, 6-alkynyl fucose, and 2,4,7,8,9-Penta-*O*-acetyl-*N*-acetyl-3-fluoro-b-d-neuraminic acid methyl ester were purchased from Carbosynth, San Diego, CA, USA.

### 2.2. Cell Line Culture

Cells were obtained from the American Type Culture Collection (ATCC, Manassas, VA, USA) and cultured in their respected media supplemented with 10% fetal bovine serum and 1% penicillin incubated at 37 °C in an atmosphere of 5% CO_2_. Media were replaced every 48 h. Caco-2 cells were treated with 50, 100, 200, and 400 μM of 3FS, 2FF, 6AF, and 25, 50, 100, and 200 μM of Kif (as a positive control) once the cells reached 40% confluency. All other cells were treated with 200 μM of 3FS, 2FF, 6AF, and 100 μM of Kif. All compounds were dissolved in dimethyl sulfoxide (DMSO) with a final treatment concentration of 0.1% (*v*/*v*) for all inhibition experiments. Caco-2 was also treated with media containing 0.1% DMSO, which resulted in no difference in the profile between the 0.1% DMSO-treated cells to the control cell. After 72 h, the cells were washed with PBS and pelleted for N-glycan release or trypsin digestion and subsequent LC–MS analysis. 

### 2.3. Cell Membrane Extraction

Extraction protocols were described previously and applied here with slight modifications [24]. Cell pellets were reconstituted in a homogenization buffer containing 20 mM 4-(2-hydroxyethyl)-1-piperazineethanesulfonic acid (HEPES) (pH 7.5), 0.25 M sucrose and protease inhibitors (Calbiochem/EMD Chemicals) at a 1:100 ratio. Using a probe sonicator (Qsonica, Newtwon, CT, USA), cells were lysed with five alterations of on and off pulses in 5 and 10 s time intervals. Cellular debris and mitochondrial fractions were pelleted by centrifugation at 2000× *g* for 10 min. Supernatants were transferred to perform ultracentrifugation at 200,000× *g* for 45 min at 4 °C. Pelleted plasma membranes were then resuspended in 500 μL of 0.2 M Na_2_CO_3_ and 500 μL of nanopure water followed by two series of ultracentrifugation at 200,000× *g* for 45 min to wash off cytoplasmic and endoplasmic reticulum fractions.

### 2.4. N-Glycan Release

Enzymatic releases of *N*-glycans were adapted from a previously developed protocol where solid-phase extraction (SPE) was performed to enrich N-glycans [25]. Samples were reconstituted in 100 μL of 100 mM ammonium bicarbonate (ABC) and 5 mM DTT and heated at 100 °C for 1 min to denature proteins. Samples were cooled at room temperature followed by the addition of 2 μL of 500,000 U/mL peptide N-glycosidase F (PNGase F), microwaved at 60 °C for 10 min to accelerate *N*-Glycans release. Samples were incubated for 18 h at 37 °C to hydrolyze the *N*-Glycans. The reaction was quenched with 350 μL of water followed by ultracentrifugation at 200,000× *g* to separate the deglycosylated proteins and the *N*-glycans. *N*-glycans in the supernatant were collected, cleaned with porous graphited carbon (PGC) SPE, dried completely, and reconstituted in 30 μL of water prior to analysis with Agilent 6200 series nano liquid chromatography chip–quadrupole time-of-flight mass spectrometry (nanoLC Chip–QTOF MS). 

### 2.5. NanoLC Chip–QTOF MS Analysis

*N*-glycans analysis were described previously and applied here with slight modifications [26]. *N*-glycans were separated using a nanoLC Chip–QTOF MS with a 65 min run time, where *N*-glycans begin to elute between 15 and 35 min. *N*-glycan separation was conducted with a binary solvent system, where solvent A was composed of 3% (*v*/*v*) acetonitrile (ACN) and 0.1% (*v*/*v*) formic acid (FA) in water, and solvent B was composed of 90% (*v*/*v*) ACN and 1% (*v*/*v*) FA in water. Samples were injected into an Agilent PGC microfluidic chip, which consisted of a 40 nL enrichment and a 75 μm × 43 nm analytical column, both with a partial size of 5 μm. The gradient sequence for the run was: 0–2.5 min, 0% B; 2.5–20 min, 16% B; 20–35 min, 58% B; 35–40 min, 100% B; 40–50 min, 100% B; 50.01–65 min, 0% B with a flow rate of 0.3 μL/min. Mass range of *m*/*z* 600–2000 with spectra were measured 1.5 s per spectrum in positive ionization mode. Reference mass m/z 1221.991 were used to correct mass inaccuracies. Quantification of the cell surface glycosylation changes were determined using MassHunter software (Agilent, Santa Clara, CA, USA, version B.07.00). The software identifies molecular features and provides a chromatographic peak volume, which provides relative intensities. The intensities are used directly to provide relative abundances. *N*-Glycan tandem data were analyzed with GlycoNote (https://github.com/MingqiLiu/GlycoNote, Last accessed on 12 May 2021). GlycoNote structures were then validated manually. K-mean cluster analysis using JMP software(SAS, Cary, NC, USA, version Pro 15) was also applied to *N*-glycans results.

### 2.6. Leica TCS SP8 STED 3X Fluorescence Imaging

PNT2 cells were cultured in a 35 mm glass bottom dish (ibidi) until 20% confluency. Cells were treated with 200 μM of 6AF for 72 h at 37 °C in an atmosphere of 5% CO_2_. After 3 days, cells were fixed with 1% paraformaldehyde, “click” with a fluorescent tag 7-Azido-4-Methylcoumarin from Sigma-Aldrich with excitation at 387 nm and emission at 470 nm, and membrane stained with Cellmark dye (ThermoFisher, Waltham, MA, USA) with excitation at 522 nm and emission at 535 nm. Images were taken at VetMed Advance Imaging Facility under 100× magnification with oil submersion.

### 2.7. Trypsin Digestion

Enzymatic digestion of membrane proteins was adapted from previous procedures followed by peptide desalting using 500 mg of C18 SPE cartridges [27]. The cell pellet was sonicated with 60 μL of 8M urea for 15 min followed by the addition of 2 μL of 550 mM DTT incubated at 55 °C for 50 min. After incubation, 4 μL of 450 mM of iodoacetamide (IAA) was added and placed in the dark for 20 min. An aliquot of 420 μL of 50 mM ABC was added to each sample with the addition of 2 μg of trypsin that was reconstituted in 50 mM ABC. Samples were then incubated at 37 °C for 18 h. For proteomic analysis, peptides were desalted using C18 SPE cartridges. The peptide concentration for proteomic analysis was determined with bicinchoninic acid assay (BCA) peptide assay (ThermoFisher, Waltham, MA, USA) prior to injection in the Orbitrap Fusion Lumos nanoLC–MS/MS instrument. 

### 2.8. Proteomic Data Analysis

The Human FASTA database was acquired from UniProt. The Raw files and FASTA file were then inputted into Byonic software version v3.11.3 for proteomic analysis followed by extract ion chromatogram (EIC) using Byologic software (Protein Metrics, Cupertino, Ca, USA, version v3.11.3). Posterior error probability (PEP) smaller than 0.01, Score > 100, peptide length > 5, and 1% false discovery rate (FDR) were applied for proteomic data analysis. Multiple t tests were performed using GraphPad Prism software (GraphPad, San Diego, CA, USA version 8).

## 3. Results

**Figure 1:**
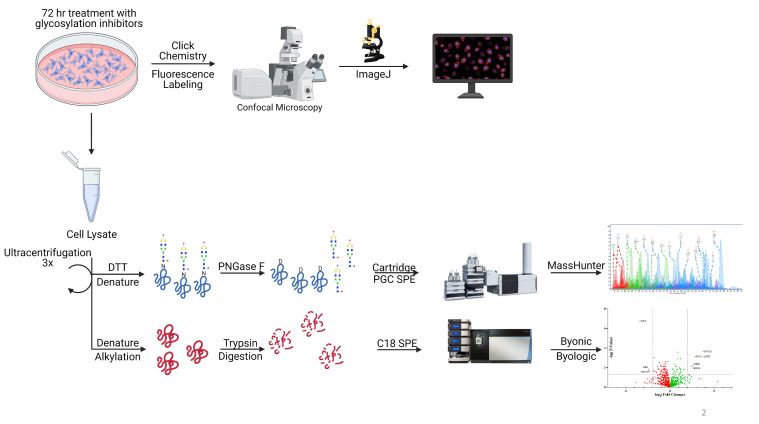
The schematic workflow for characterization of *N*-glycomics, proteomics, and confocal microscopy. Glycosylation inhibitors were added to the supernatant and treated for 72 h followed by cell membrane extraction and analysis. Click chemistry was performed after 72 h of treatment followed by imaging to determine 6-alkynyl fucose (6AF) incorporation. Schematic workflow was created with BioRender.com last accessed 17 August 2021.

The workflow for the glycomic characterization of the cell lines is shown schematically in Figure 1. It involves the harvesting and lysing of the cells followed by a series of ultracentrifugation to enrich the cell membrane. The enriched fractions are subjected to *N*-glycan release using the enzyme PNGase F. NanoLC–MS using a PGC stationary phase and QTOF mass analyzer produces a glycan profile with isomer separation. In the *N*-glycomic profile, we generally identify over 300 structures. The membrane enrichment has been validated in previous publications [28]. 

### 3.1. N-Glycan Profile of Cell Membranes 

**Figure 2:**
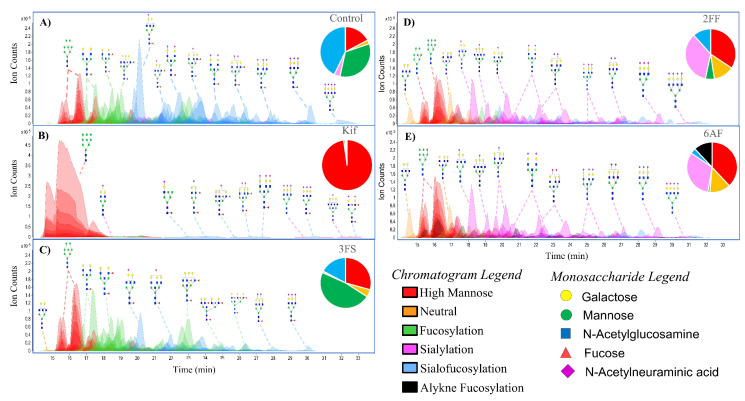
Extracted compound chromatograms of Caco-2, showing (**A**) control, (**B**) Kifunensine (Kif), (**C**) 2,4,7,8,9-Penta-O-acetyl-*N*-acetyl-3-fluoro-b-d-neuraminic acid methyl ester (3FS), (**D**) 2-deoxy-2-fluorofucose (2FF), and (**E**) 6-alkynyl fucose. Peaks are colored by glycan subtypes and annotated with the schematic representation of the glycan structures. Monosaccharide notations follow the Symbol Nomenclature for Glycans (SNFG) system [29] and N-glycans were drawn with GlycoWorkbench version 2.1 [30].

The *N*-glycome profile for Caco-2, a colon epithelial carcinoma line, was determined and yielded over 200 glycans (including isomers) (Figure 2A). The untreated cells yielded glycans composed of high-mannose- (18.0%), complex- (47.3%), and hybrid-type structures (34.7%). With regard to the complex glycans, they were composed of mono- (0.3%), bi- (2.0%), tri- (6.5%), and tetra-antennary (29.5%) structures. Other structural features included bisecting *N*-acetylglucosamine (GlcNAc) (9.0%). The complex and hybrid compounds were further separated into sialylated (3.8%), fucosylated (33.7%), and sialofucosylated (42.7%). The sialylated glycans were mainly singly (42.6%), doubly (12.9%), triply (3.5%) and quadruply sialylated (0.4%) glycans. Similarly, the fucosylated glycans were composed of singly (36.7%), doubly (24.8%), triply (13.2%), quadruply (6.9%) and quintuply fucosylated (1.1%) glycans. These results were consistent with previously published values for the undifferentiated Caco-2 cell line, which similarly yielded significantly higher amounts of complex-type structures with high levels sialofucosylation. There were over 300 structures composed of nearly 100 compositions with only 16 representing nearly 50% of the intensities (Supplementary Figure S1). The most abundant composition was Hex_5_HexNAc_5_Fuc_1_NeuAc_1_. 

The inhibitors were introduced to the media with 0.1% DMSO. To ensure that DMSO had little effect on the cell surface glycan expression, a comparison of DMSO-treated cells with untreated cells was performed. The difference was barely observed (Figure S2A), which suggested that DMSO had no effect and did not alter the membrane glycome. To determine the reproducibility, triplicate experiments were performed. Supplementary Figure S2B shows the *N*-glycan profiles of Caco-2 cells treated with 2FF inhibitor performed in triplicates. The coefficient of variation (CV) for compounds varied less than 20%. The variations in the *N*-glycan subtypes averaged were below 20%. 

The effect of the inhibitor concentration was monitored. Concentrations between 25 and 400 µM were introduced to the supernatant and allowed to incubate for several hours. The glycan profiles were obtained for each experiment. For Kif, 100 µM concentrations and 72 h incubation proved optimal. For the other inhibitors, higher concentrations were needed at 200 µM and 72 h (Supplementary Figure S3). 

The addition of Kif produced the largest effect on the glycocalyx. The LC–MS chromatogram yielded primarily high mannose structures (over 95% in total abundances) (Figure 2B). The remaining compounds in abundances are residual complex-type *N*-glycans. In comparison, the untreated cells contained only 18.0% high mannose structures. 

The compound 3FS is a known sialyltransferase inhibitor. The sialic acid analog is incorporated into the salvage pathway and converted to the active form of the sugar by attaching cytidine-5′-monophosphate (CMP), resulting in the nucleotide sugar CMP-3FS [31,32]. The active form of the sugar then inhibits the transferase, depleting the cells of sialylated glycans. Figure 2C shows the *N*-glycan profile for Caco-2 treated with 3FS. Firstly, there was no incorporation of 3FS into the cell surface *N*-glycome. The 3FS-treated Caco-2 cells were composed of high mannose (29.9%), complex (37.5%), and hybrid-type (32.6%) structures. Sialylated and sialofucosylated glycans decreased by 28% while fucosylated glycans increased by 14% compared to the untreated cell. The increase in fucosylated glycans is due to the loss of sialylated decoration in the previously sialofucosylated species. For example, the compound Hex_5_HexNAc_5_Fuc_1_ increased from 3.3% to 6.0% upon treatment with 3FS. The increase is caused by the loss of sialic acids from the three sialylated analogs, namely, Hex_5_HexNAc_5_Fuc_1_NeuAc_1_, Hex_5_HexNAc_5_Fuc_1_NeuAc_2_, and Hex_5_HexNAc_5_Fuc_1_NeuAc_3_. However, it should be noted that the total loss of sialylated glycans was not observed. Among the fucosylated species, Hex_5_HexNAc_5_Fuc_1_NeuAc_1_ was still the most abundant sialofucosylated glycan. 

The fucose transferase inhibitor 2FF is converted with guanosine 5′-diphosphate (GDP) to GDP-2FF through the fucose salvage pathway [33]. This active sugar inhibits fucosyltransferases and led to the profile shown in Figure 2D. We examined the glycan profile and determined that 2FF was similarly not incorporated into the N-glycans. The N-glycome was composed of 34.7% high mannose, 39.7% complex, and 25.6% hybrid. Fucosylated glycans decreased from 76% to 17% after 2FF treatment. An increase in the sialylated glycans was observed from 3.8% to 35.1%. For example, the structure Hex_5_HexNAc_5_NeuAc_1_ increased from 0.5% to 6.5% likely due to the decrease in fucosylated species, including Hex_5_HexNAc_5_Fuc_1_NeuAc_1_, Hex_5_HexNAc_5_Fuc_2_NeuAc_1_, and Hex_5_HexNAc_5_Fuc_3_NeuAc_1_. An important observation in comparing the two inhibitors, 2FF and 3FS, was that they appeared to behave independently. It seems that the decoration of fucose and the decoration of sialic acid on sialofucosylated glycans are not dependent on one another.

The compound 6AF was known to inhibit the de novo pathway through interactions with 3,5-epimerase-4-reductase, thereby diminishing fucose incorporation. This pathway converts mannose into fucose, forming the activated reagent GDP-fucose [18]. Unlike the other inhibitors, examination of the N-glycome profile showed that 6AF was incorporated into specific glycans (Figure 2E). The N-glycome profile yielded 38.1% high mannose, 37.7% complex, and 24.2% hybrid. Complex and hybrid glycans were composed of 1.2% fucosylated, 31.8% sialylated, and 3.6% sialofucosylated. An additional 11.9% was obtained for the 6AF incorporated species corresponding to 6AF-fucosylated and 6AF-sialofucosyalted glycans combined. In general, 6AF behaves similarly to 2FF in that total fucosylation was decreased. However, incorporation of the inhibitor was also observed for 6AF. 

**Figure 3:**
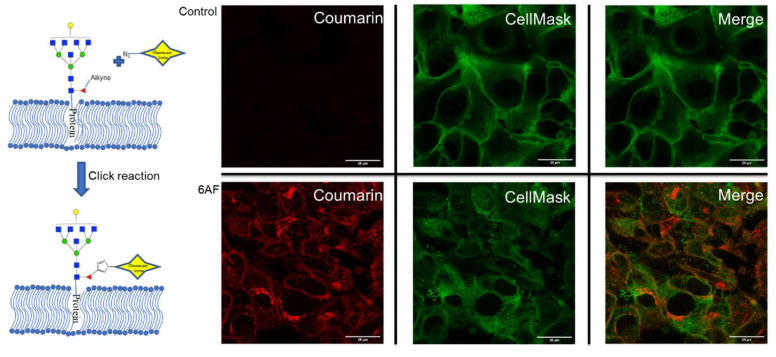
Confocal microscopy of PNT2 cells were the top cells without 6AF treatment (control) and the bottom cells with 6AF treatment. Cells were stained with CellMask™ (green) and 7-Azido-4-Methylcoumarin (red) (ThermoFisher, Waltham, MA, USA). The merge section is the overlap between CellMask™ and 7-Azido-4-Methylcoumarin.

The incorporation of 6AF was further confirmed by MS/MS analysis (Supplementary Figure S4). However, no attempt was made to differentiate between core and antennary fucosylation. It is noted that only one 6AF was incorporated at most, with the most abundant being Hex_6_HexNAc_7_6AF_1_NeuAc_3_. For PNT2, the inhibition created a nearly 40% drop in fucosylation, with the remaining fucosylated species containing between 20 and 30% 6AF and the rest with native fucose. To validate the incorporation, we probed cell lines with Click chemistry using a chromophoric tag. A control and 6AF-treated PNT2 cells were reacted for one hour with 7-Azido-4-Methylcoumarin followed by staining with CellMask™ Deep Red (cell plasma membrane). The merged images of 6AF and CellMask™ confirmed the incorporation of 6AF both on the cell plasma membrane and Golgi (Figure 3). The incorporation of 6AF under similar conditions was reported previously [17]. The inhibitor should therefore be used with caution in assessing fucose function.

**Figure 4:**
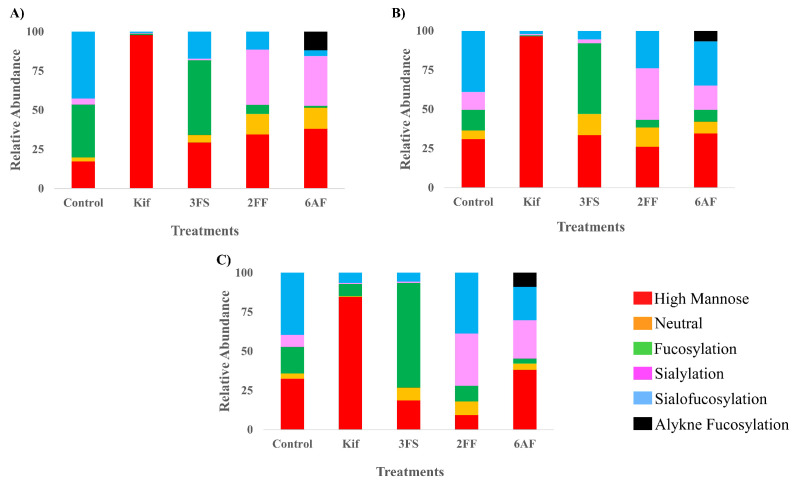
The relative abundance profile of each glycosylation inhibitor with its control counterpart plotted on stack columns with (**A**) Caco-2, (**B**) A549, and (**C**) PNT2 cell lines. Stack column plots are color coded by glycan subtypes.

To observe the general behavior of the inhibitors, each one was similarly examined with additional cell lines, namely, A549 (lung epithelial carcinoma) and PNT2 (normal prostate epithelial). These cell types were chosen to study the effect of each inhibitor on different cell types. The comparison of the results for Caco-2, A549, and PNT2 (Figure 4) was presented according to their major glycan types. A549 and PNT2 behaved similarly when treated with Kif yielding high mannose glycans with abundances of 96% and 85%, respectively. When treated with 3FS, the cells lost most of their sialylated glycans, with A549 having only 8% sialylated glycans after treatment compared to 50% before, which is very similar to PNT2 with 6% compared to 47%. Fucosylated glycans in PNT2 cells treated with 2FF decreased by 7%, while A549 decreased by 24%. PNT2, when treated with 6AF, lost more fucosylated glycans compared to 2FF with 33% loss and a 9% incorporation of 6AF. No incorporation of 2FF was observed in any of the cell lines. A549 lost 17% of its fucosylated glycans with a 7% incorporation of 6AF. 

K-mean clustering analysis was performed on the inhibition dataset with all three cell lines. K-mean clustering is a type of unsupervised machine learning where random centroids are generated. The algorithm then calculates the Euclidian distance between a point and the nearest centroid. Afterward, all the points nearest to the centroid are averaged and the centroid position is shifted to the average distance. This is done for all centroids, and it iterates until convergence. Through this process, each treatment was clustered together with one another regardless of which cell lines were being treated (Supplementary Figure S5). The orange cluster contains cells treated with Kif. This cluster had the tightest pack, suggesting that the inhibition effects were most similar to each other regardless of the cell type, while 6AF-treated cells (light green cluster) had the most variation between the cells based on the spacing in the clustering. Though each cell line had slight variation in the results, the overall effect of the inhibitors was independent of the cell itself. 

### 3.2. Cell Surface Proteome with Inhibitors

**Figure 5:**
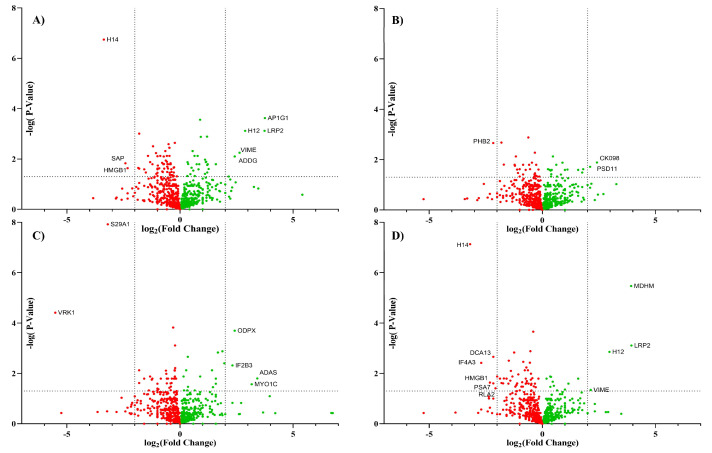
The volcano plot represents the proteomic data from Caco-2 comparing control to an inhibition treatment including (**A**) Kif, (**B**) 3FS, (**C**) 2FF, and (**D**) 6AF. Each data point represents a protein with the downregulation (red) and upregulation (green).

To determine whether the glycan modification by the inhibitors altered protein expression, proteomic analysis was performed on enriched cell membrane. The list of proteins associated with each treatment was provided in the Supplementary Information. A volcano plot (Figure 5) was constructed to determine the similarities between the proteins in each treatment group compared to the untreated control group. 

Biological triplicates of Caco-2 cells treated with each inhibitor were compared to untreated triplicate cells using raw XIC values extracted from Byologic software. The resulting data were sorted by peptide length less than 5 and score less than 100 to remove potential missed cleavages sequence. Each protein was represented by the most abundant peptide signal. The thresholds for significant proteins were p-values less than or equal to 0.05 and log_2_ fold change less than -2 or greater than 2.

Among all the treatments, the largest change in the glycomic profile was observed by the addition of kifunensine. Kif converted the glycans from complex-type structures to high mannose structures with the major abundance corresponding to Man_9_. Proteomic analysis of both Kif-treated cells and the control yielded around 1100 proteins for each treatment. There were over 1000 proteins that were conserved in both groups, and 9 proteins were identified as significant using the above criteria. Among those nine proteins, only five were cell surface proteins. The proteomic results show little variations in the proteins identified between the control and Kif-treatment. Based on these results, there is apparently little change in protein expression even with large dramatic changes in glycan expression, suggesting that glycosylation does not appear to affect the membrane protein composition significantly.

The comparison of the other treatments yielded similar results with minor variations between the treatments. They all yielded around 1100 proteins, with approximately 1000 proteins conserved between the treated cells and the untreated control cells. Inhibiting sialylation with 3FS or inhibiting with fucosylation yielded a smaller change in the membrane protein expression compared to Kif. On the other hand, 6AF inhibition yielded more significant numbers of proteins than Kif-treated cells using the above criteria. There were 17 proteins that were determined to be significant in 6AF-treated cells. Even so, only a handful of proteins were determined to be cell surface proteins (LRP2, VIME, and HMGB1). This further suggests that the inhibition of cell surface glycosylation had little effect on protein expression.

## 4. Discussion

Glycomic profiling provides an extensive characterization of metabolic inhibitors and their efficacy. The efficacy of each inhibitor varied. Among them, Kif had the highest efficacy and was capable of removing all other glycan types besides high mannose type. Kif is known to inhibit mannosidase I (Man 1A1), the enzyme necessary to trim high mannose structures and produce complex- and hybrid-type structures [34]. Thus, the action of this compound primarily yields the Man9 glycan (Hex_9_HexNAc_2_). The conversion was high, up to 97% high mannose. There were remaining complex-type structures in, for example, Caco-2, resulting in some 3% remaining sialofucosylated glycans. In general, none of the inhibitors yielded complete conversion, at least as detected by the method. There could be several factors that can leave residual glycans, with the most likely cause to be differences in protein turnover associated with the glycoform. We have previously shown that proteins on the cell membrane express differential turnover rates [12], with sialylated proteins having the lowest rates. Indeed, the remaining were generally complex-type glycans with sialylation. In general, sialylation is more difficult to suppress due the slower turnover and the greater number of sialyltransferase.

Cells treated with the sialic acid inhibitor 3FS decreased sialylation by approximately 60%. The sialic acid analog is incorporated into the salvage pathway and converted to the active form of the sugar by attaching CMP resulting in the nucleotide sugar CMP-3FS [31,32]. The active form of the sugar then inhibits the transferase, depleting the cells of sialylated glycans. Cells treated with fucose inhibitors 2FF and 6AF decreased fucosylated species by nearly 80%. 6AF had the additional complication of incorporating into the glycan. The efficacy of each inhibitor was consistent regardless of cell type shown in the N-glycome profile of different cell types and K-mean clustering analysis. Additionally, Caco-2 proteomic results indicated that each inhibitor had little effect on the proteins expressed on the cell surface membrane for this cell line. The results strongly suggest that altered glycosylation does not affect protein expression in the glycocalyx of Caco-2. Kif had the highest efficacy, but less than one percent of the cell surface proteomic showed a significant change in protein expression.

In summary, we were able to profile the effect of these glycosylation inhibitors on the glycocalyx using a nanoLC-chip–QTOF MS, overcoming the limitation of other non-structurally specific methods. Through this method of analysis, we were able to obtain a more qualitative analysis of each inhibitor’s effect to give a more in-depth understanding of how each inhibitor alters glycosylation. Future studies can use this application to study the functional role of different decorations of glycosylation in other biological systems.

## Data Availability

Data generated for this manuscript are included in the main figures or Supplementary Materials figures. The *N*-glycomic MS data can be found in GlycoPOST and the proteomic MS data can be found in PIRDE.

## Supplementary Materials

The following are available online at https://www.mdpi.com/article/10.3390/cells10092318/s1.
