# Supplementary material for: N-Glycomic Analysis of the Cell Shows Specific Effects of Glycosyl Transferase Inhibitors
Source: Cells. 2021 Sep 4;10(9):2318. doi: 10.3390/cells10092318 (PMC8465854; doi:10.3390/cells10092318)
Supplement: Supplementary file 1 [file cells-10-02318-s001.zip › cells-1329512-supplementary.pdf]

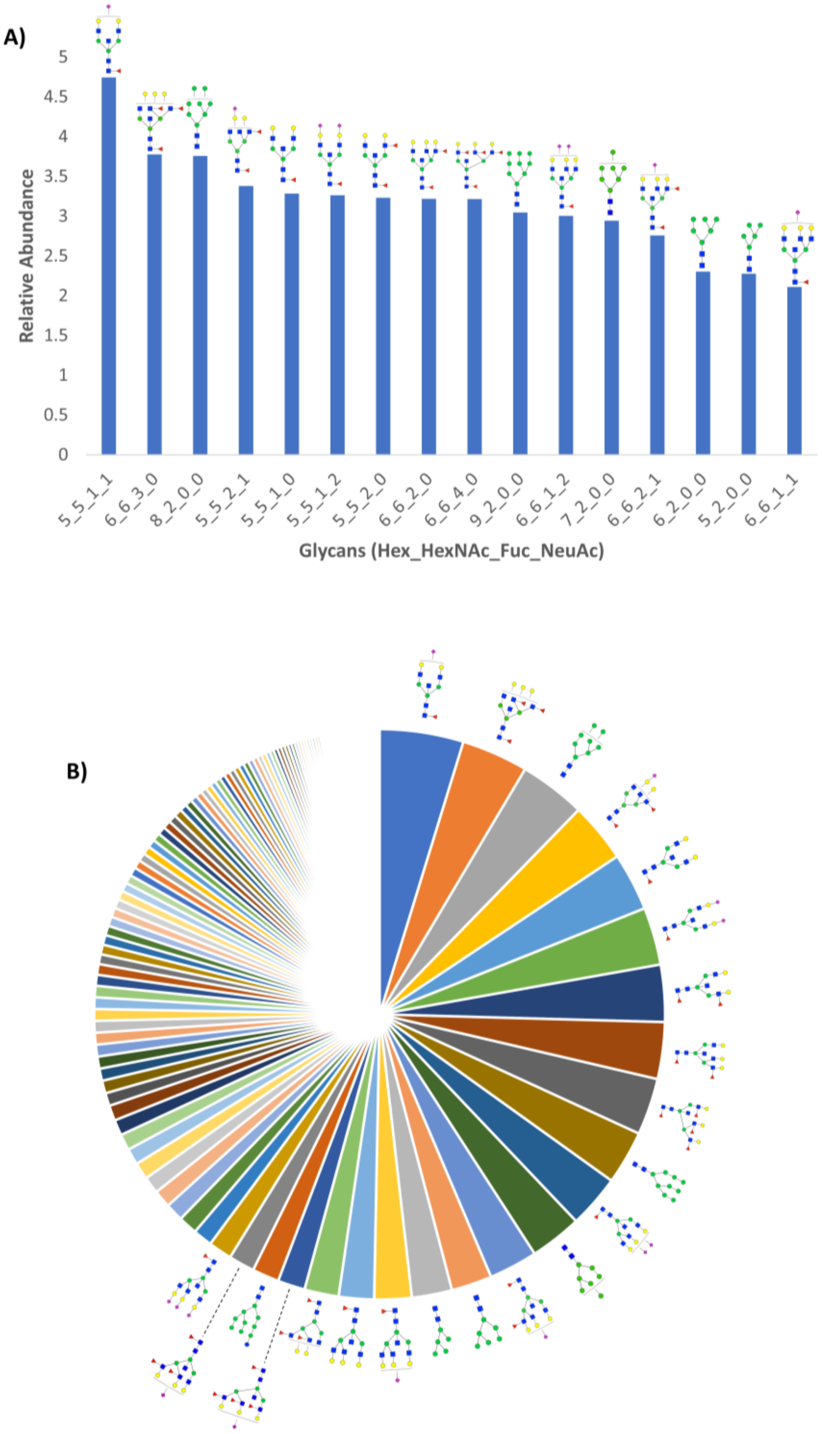

Figure S1. Relative abundant N-glycans found in Caco-2 (A) the most abundant glycans and (B) are all the N-glycan identified with MassHunter software. N-Glycans were drawn with GlycoWorkbench [30].

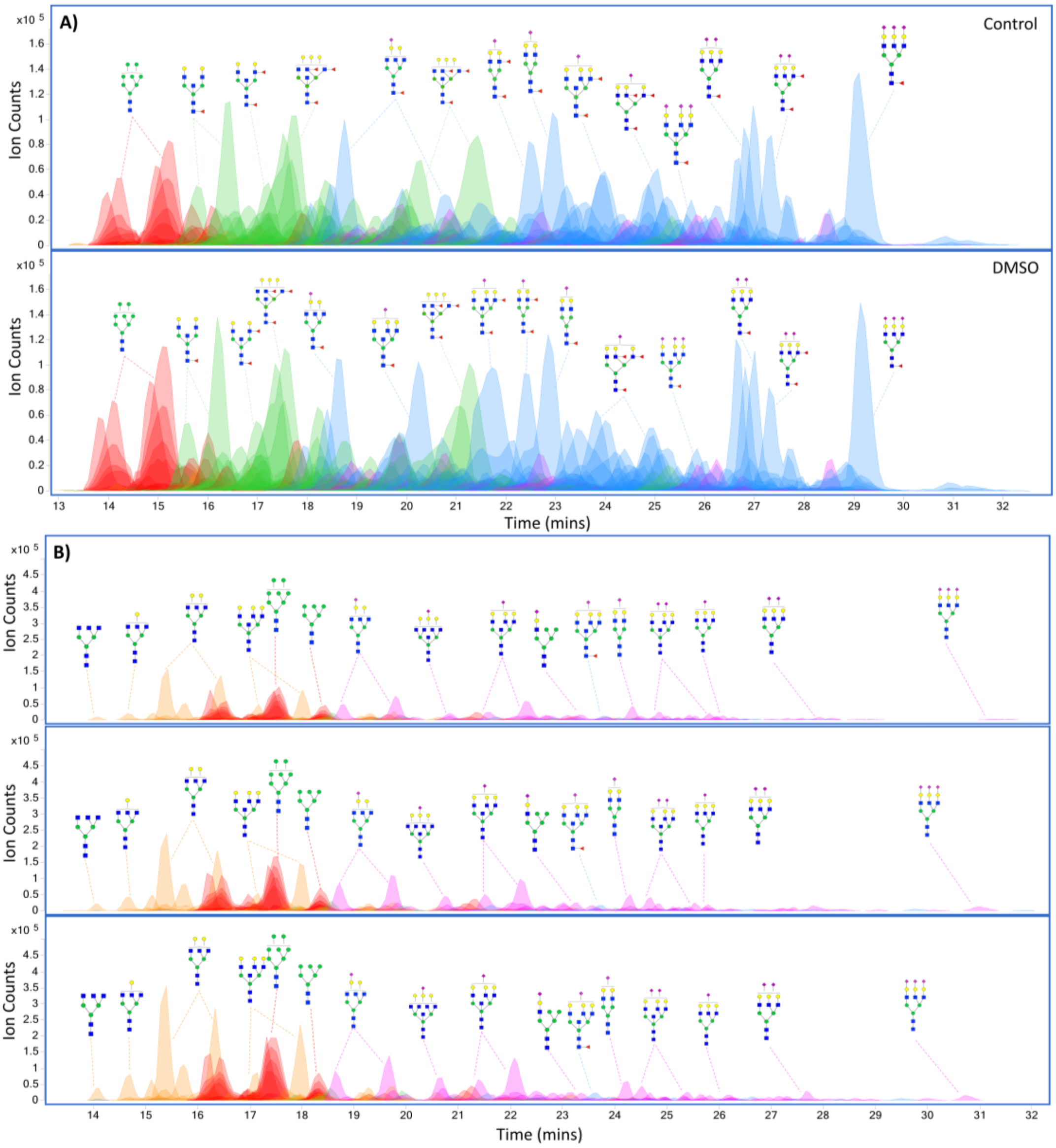

Figure S2. Extracted compound chromatograms of Caco-2, showing (A) comparing untreated control to DMSO treated cells, (B) biological replicates of Caco-2 treated with 2-deoxy-2-fluorofucose. Peaks are colored by glycan subtypes and annotated with the schematic representation of the glycan structures. Monosaccharide notations follow the Symbol Nomenclature for Glycans (SNFG) system [29] and N-Glycans were drawn with GlycoWorkbench [30].

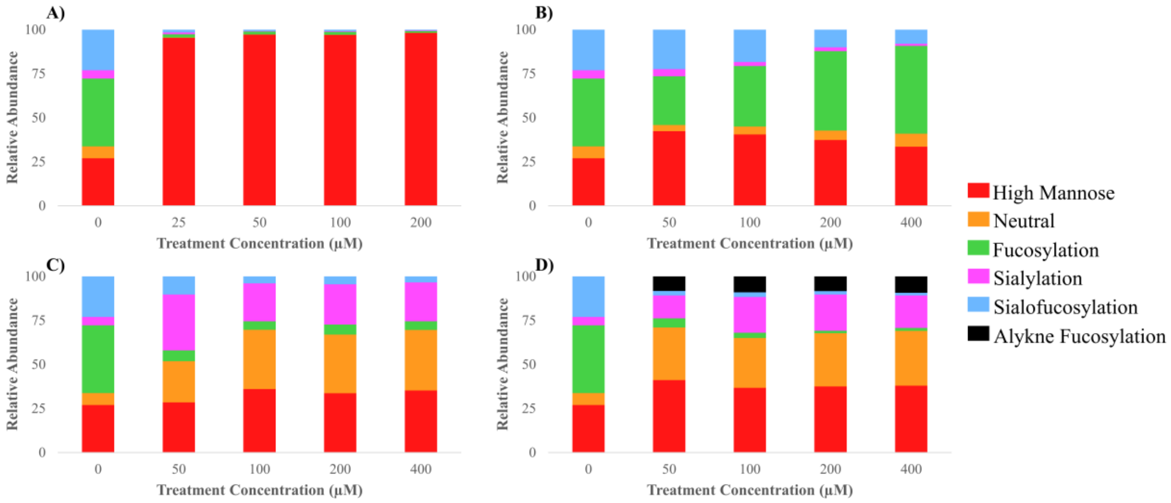

Figure S3. The relative abundance of Caco-2 with different concentration of glycosylation inhibitors, showing (A) Kifunensine, (B) 2,4,7,8,9-Penta-O-acetyl-N-acetyl-3-fluoro-b-D-neuraminic acid methyl ester, (C) 2-deoxy-2-fluorofucose, and (D) 6-alkynyl fucose. Concentration ranges from 25μM to 400μM and in each stack column plot are color coded by glycan subtypes.

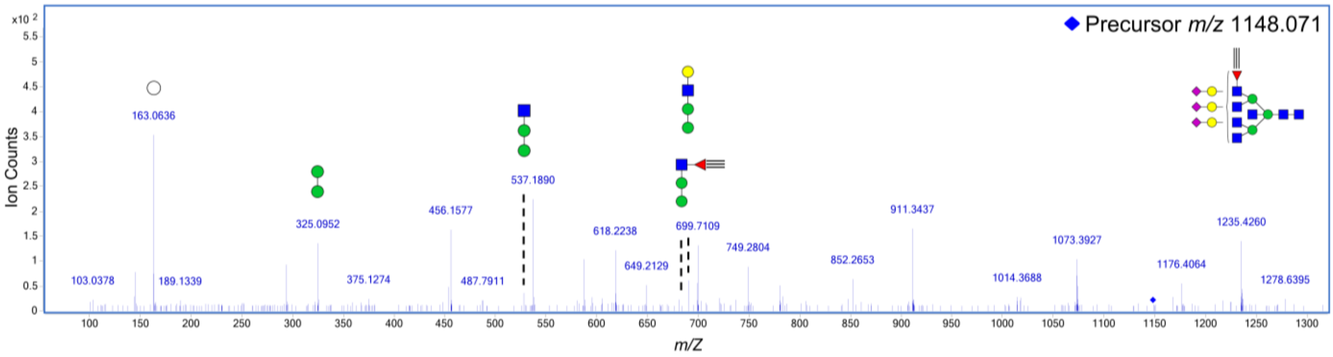

Figure S4. N-Glycomic MS/MS spectra of PNT2 treated with 6AF. This exhibits 6AF incorporation in $Hex_6HexNAc_76AF_1NeuAc_3$. Monosaccharide notations follow the Symbol Nomenclature for Glycans (SNFG) system [29] and N-Glycans were drawn with GlycoWorkbench [30].

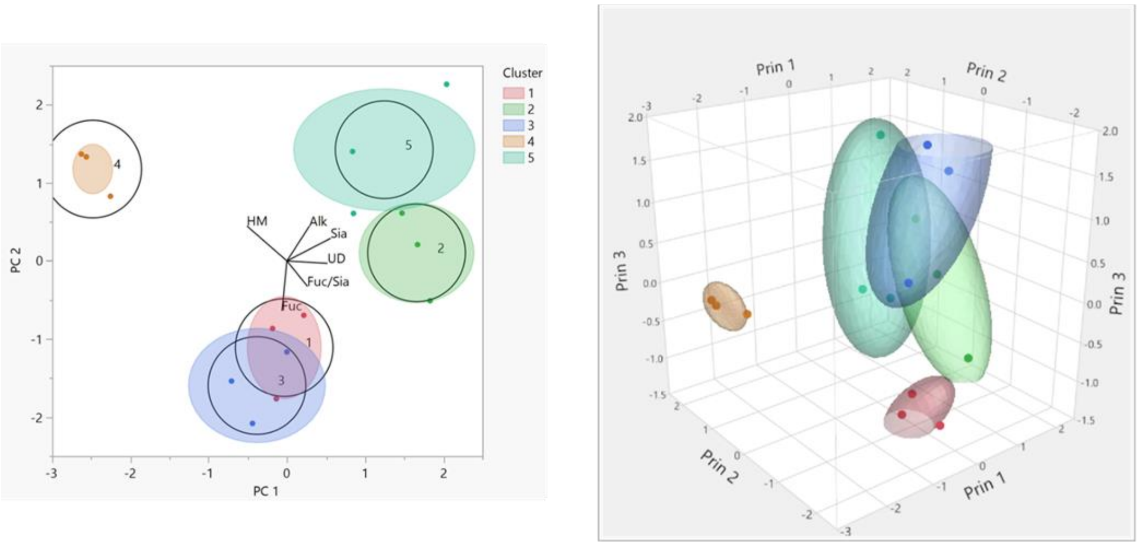

Figure S5. Two-dimensional and three-dimensional k-mean cluster of the relative abundance of each cell line (Caco-2, A549, and PNT2) with and without inhibition treatments (Kifunensine, 2,4,7,8,9-Penta-O-acetyl-N-acetyl-3-fluoro-b-D-neuraminic acid methyl ester, 2-deoxy-2-fluorofucose, and 6-alkynyl fucose. Red cluster 1 (untreated control cells), dark green cluster 2 (2FF treated cells), dark blue cluster 3 (3FS treated cells), orange cluster 4 (Kif treated cells) and light green cluster 5 (6AF treated cells). The plot was created with JMP Pro 15 software using K-mean clustering analysis.
